# New insights into the phylogeny and taxonomy of Chinese *Physospermopsis* (Apiaceae)

**DOI:** 10.3897/phytokeys.175.57681

**Published:** 2021-03-23

**Authors:** Xin-Rui Xu, Xian-Lin Guo, Megan Price, Xing-Jin He, Song-Dong Zhou

**Affiliations:** 1 Key Laboratory of Bio-Resources and Eco-Environment of Ministry of Education, College of Life Sciences, Sichuan University, 610065, Chengdu, Sichuan, China Sichuan University Chengdu China; 2 Sichuan Key Laboratory of Conservation Biology on Endangered Wildlife, College of Life Sciences, Sichuan University, 610065, Chengdu, Sichuan, China Sichuan University Chengdu China

**Keywords:** Apiaceae, morphology, phylogeny, *
Physospermopsis
*, taxonomy

## Abstract

*Physospermopsis* (Apiaceae) comprises about 10 species, but its taxonomy and phylogeny are disputed. The genus is mostly distributed in the Himalayas and Hengduan Mountains at high elevation. Earlier molecular studies involving six species of *Physospermopsis* indicated that this genus is not monophyletic and is nested in the East Asia Clade. Therefore, the aims of this study were to re-assess the phylogenetic position of, and interspecific relationships within, *Physospermopsis* based on two chloroplast loci (*rpl16*, *rps16*) and one nuclear region, the internal transcribed spacers of ribosomal DNA (ITS). Eight species involving 13 populations of *Physospermopsis* were collected. These were sequenced and analyzed with the sequences of 31 other Apiaceae species obtained from the NCBI to determine phylogenetic relationships using Bayesian inference (BI) and Maximum likelihood (ML). Our study found that *Physospermopsis* is monophyletic, nested in Pleurospermeae of Apiaceae, sister to *Pleurospermum*. And we propose that the *Physospermopsis* clade should be replaced by the East Asia Clade. However, the interspecific relationships within *Physospermopsis* were not well resolved and the positioning of species was unclear. Diagnostic characteristics to distinguish *Physospermopsis* species in the field and laboratory are provided for future *Physospermopsis* phylogenetic studies.

## Introduction

*Physospermopsis* H. Wolff (1925: 276) has been reported to contain about 10 species, with eight species distributed in China (Pan and Watson 2005). However, 11 species were reported by Pimenov and Kljuykov (Pimenov and Kljuykov 2000a, b, c; Pimenov 2017). There has been difficulty in interpretation of diverse morphology to diagnose species and even limits of the genus. Most species of this genus occur in the Himalayas and Hengduan Mountains, and of these, four are endemic to the Hengduan Mountains (Wang and Pu 1992). In China, most species of *Physospermopsis* grow in open forests, scrubs, grasslands and alpine meadows at elevations of 2250–4800 m (Wolff 1929; Mukherjee 1982; Farille and Malla 1985; Pan and Watson 2005). *Physospermopsis* is characterized by having a long, conic taproot, ribbed stem, pinnate, rarely entire leaf blade, prominent, leaf-like bracts, variable bracteoles, minute calyx teeth, emerald young fruits, ovoid to broadly ovoid mature fruit with slightly cordate base (Wolff 1925; Pan and Watson 2005). Based on an analysis of previous research (Wang and Pu 1992; Pu and Liu 2005, 2006), *Physospermopsis* species usually possess a pericarp with wavy stria or reticulate ornamentation, prominent or inconspicuous fruit ribs, diverse carpoderms and endosperms, and pollen morphology showing a trend from rhomboidal type to rectangular type.

Previous studies on *Physospermopsis* have been extensive, including on micromorphology, anatomy and pollen morphology (Wang and Pu 1992; Pu and Liu 2005, 2006). However, previous molecular phylogenetic analyses of *Physospermopsis* have only involved a small number of taxa mostly using internal transcribed spacer (ITS) sequences (Downie et al. 2000; Calviño et al. 2006; Zhou et al. 2008, 2009; Downie et al. 2010; Valiejo-Roman et al. 2012). Phylogeny of *Physospermopsis* has been disputed with Downie et al. (2000) placing *P. kingdon-wardii* (H.Wolff) C.Norman (1938: 231) and *P. rubrinervis* (Franchet) C.Norman in the *Komarovia* clade based on the materials collected from Yunnan, China. Then, Calviño et al. (2006) provisionally positioned *Physospermopsis* in the *Physospermopsis* clade based on more comprehensive maximum parsimony (MP) analyses of ITS sequences, which arose as a weakly supported sister group to the *Komarovia* clade. Later, Zhou et al. (2008) studied five species of *Physospermopsis* and concluded that *Physospermopsis* was not a monophyletic group. Zhou et al. (2008) placed *P. kingdon-wardii* and *P. rubrinervis* in the East Asia clade and referred them to *Trachydium* J.Lindley (1835: 232). Zhou et al. (2008) also concluded that *P. cuneata* H.Wolff (1929: 126) was nested in Pimpinelleae and should be close to *Pimpinella* C.Linnaeus (1753: 263), while *P. muliensis* R.H.Shan & S.L.Liou (1979: 105) and *P. shaniana* C.Y.Wu & F.T.Pu (1993: 1285) were allied within Pleurospermeae. Additionally, the East Asia clade was proposed as the synonym of *Physospermopsis* clade due to almost all of its species being primarily distributed in East Asia (Zhou et al. 2008). The following year, Zhou et al. (2009) added *P. delavayi* (the nomenclatural type of *Physospermopsis*) to their previous analyses and placed it in Pleurospermeae. Downie et al. (2010) decided that *Physospermopsis* should be placed in the *Physospermopsis* clade (East Asia clade) and was not monophyletic, but did not include the type species in their analyses potentially influencing their conclusions. Valiejo-Roman et al. (2012) conducted a molecular phylogenetic analysis of the genus *Pleurospermum* G.F.Hoffmann (Hoffmann 1814) and its related genera, including three *Physospermopsis* species.

*Physospermopsis* is a taxonomically complex genus whose generic limits with *Pleurospermum*, *Tongoloa* H.Wolff (1925: 279), and *Trachydium* are problematic (Pan and Watson 2005). Therefore, misidentification was common due to the absence of convincing morphological evidence, limitations of collected materials and examinations of type specimens. Additionally, until now there has been no comprehensive analysis using molecular phylogenetics and morphology within the one study. Therefore, we aimed to determine an accurate phylogeny of *Physospermopsis* and infrageneric relationships within *Physospermopsis* based on molecular, morphology data and combined analysis linking phylogeny and morphology. We acquired accurate data by collecting field specimens of eight *Physospermopsis* species involving 13 populations from their type localities and adjacent areas. Species were identified by field observations, validation with herbarium specimens and primary literature.

## Material and methods

### Specimen examinations, field investigations and morphology observations

The taxonomic identification of *Physospermopsis* species was by field investigations and specimen examinations from herbaria BM, BNU, CDBI, CVH, E, HITBC, ILL, K, KUN, LBG, LE, MW, NAS, NHW, P, PE, PEY, SABG, SM, SZ, UC, WU, WUK.

In the field investigations, we sampled three populations of *P. delavayi*, two populations of *P. rubrinervis*, two populations of *P. shaniana*, one population of *P. obtusiuscula* (1938: 231) and one population of *P. nana* (2000: 538) in Yunnan Province. We sampled one population of *P. kingdon-wardii* and one population of *P. obtusiuscula* in Tibet. One population of *P. alepidioides* (H.Wolff & Hand.-Mazz.) R.H.Shan (1941: 187) and one population of *P. muliensis* were sampled in Sichuan Province. All populations were collected from the type locality and adjacent regions, and the features of every species were closely matched with the types and original descriptions (de Candolle 1830; Franchet 1894; Diels 1912; Wolff 1929; Wolff et Handel-Mazzetti 1933; Shan et Liou 1979). The specific collection information are listed in Appendix 1.

Fruits, leaf segments and specimens from these eight species of *Physospermopsis* were collected in the field for morphological study. Morphological analyses of leaves and fruits based on herbarium specimens or formaldehyde-acetic acid-alcohol (FAA) preserved material were photographed by a stereomicroscope Nikon SMZ25 (Japan). The morphological data were measured using KaryoType (Altnordu et al. 2016).

### Taxon sampling

We sampled 13 populations, representing eight species of *Physospermopsis* in our phylogenetic analysis, and obtained 31 sequences of other Apiaceae species from the NCBI (Appendix 1). Based on previous research (Zhou et al. 2009), *Bupleurum krylovianum* B.K.Schischkin (1935: 2010) and *Bupleurum rockii* H. Wolff (1929: 187) were selected as the outgroup for studying the phylogenetic position of *Physospermopsis*. We chose *Pl. franchetianum* W.B.Hemsley (1892: 307) and *Pl. wrightianum* H.Boissieu (1903: 847) as the outgroup for studying interspecific relationships within *Physospermopsis*. The DNA sequences of two chloroplast loci (*rpl16*, *rps16*) and one nuclear region, the internal transcribed spacers of ribosomal DNA (ITS), were used for phylogenetic analyses. According to the research to date (Zhou et al. 2008, 2009; Downie et al. 2010; Guo et al. 2018; Panahi et al. 2018), these three markers should be sufficient to obtain the general information about relationships within the genus and its phylogenetic position within the family Apiaceae.

### DNA extraction and sequencing

The fresh leaves were collected from field specimens in Yunnan, Sichuan and Tibet, China. Voucher specimens were deposited in the Herbarium of Sichuan University (SZ). Total genomic DNA was extracted from silica-dried leaves with plant genomic DNA kit (Cwbio Biosciences, Beijing, China). The universal primers ITS4 (5’-TCC TCCGCT TAT TGA TAT GC-3’) and ITS5(5’-GGA AGT AAA AGT CGT AAC AAG G-3’; White et al. 1990) were used to amplify the entire internal transcribed sequences. The *rpl16* intron region was amplified using primers F71(5’-GCT ATG CTT AGT GTG TGA CTC GTT G-3’) and R1516 (5’-CCC TTC ATT CTT CTA TGT TG-3’) (Jordan et al. 1996; Kelchner and Clark 1997). The *rps16* sequences were amplified with primers *rps16* 3’exon (5’-CCT GTA GGY TGN GCN CCY TT-3’) and *rps16* 5’exon (5’-AAA CGA TGT GGN AGN AAR CA-3’)(Downie and Katz-Downie 1999). PCR amplification was implemented in a 30 μL volume reaction, including 3 μL total DNA, 1.5 μL forward primer, 1.5 μL reverse primer, 15 μL 2×Taq MasterMix (Cwbio, Beijing, China), and 9 μL ddH2O. The amplification of the ITS region was obtained by initial denaturation for 3 min at 94 °C, followed by 30 cycles of 45 s at 94 °C, 70 s at 54 °C, and 90 s at 72 °C, then final extension of 10 min at 72 °C. Amplification of cpDNA intron regions was obtained by initial denaturation for 3 min at 94 °C, followed by 36 cycles of 45 s at 94 °C,70 s at 58.5 °C, and 90 s at 72 °C, then final extension of 10 min at 72 °C. All PCR products were separated using a 1.5% (w/v) agarose TAE gel and sent to Sangon (Shanghai, China) for sequencing. New sequences obtained for this study have been deposited in GenBank. GenBank accession numbers are provided in the Appendix 1.

### Data analysis

We used SegMan7 (Burland 2000) to assemble ITS and cpDNA sequences. ClustalX (Jeanmougin et al. 1998) was used to align DNA sequences with manual adjustment. We then used MEGA7 (Kumar et al. 2016) to manually adjust and obtain ITS and cpDNA datasets. Gaps were positioned to minimize nucleotide mismatches. Bayesian inference (BI) and Maximum likelihood (ML) methods were used for phylogenetic analyses, using MrBayes v3.2 (Ronquist et al. 2012) and RAxML v8.2.4 (Stamatakis 2014), respectively. Before undertaking BI analyses, MrModeltest version 2.2 (Nylander 2004) was used to determine the best model of nucleotide substitution and the GTR+G model under the Akaike Information Criterion (Akaike 1974) was selected. Bayesian analyses were performed over 20 million generations with a variant of Markov Chain Monte Carlo (MCMC) method and the trees were saved to a file every 1,000 generations. The first 20% trees were discarded as “burn-in” and the remaining 80% trees were used to build a majority-rule consensus tree based on analysis of the program Tracer v1.4 (Drummond and Rambaut 2007). ML analyses were performed using RAxML v8.2.4 with the GTR+G model and 1,000 bootstrap replicates. We constructed the BI tree with ITS data from all 44 taxa to test the systematic position of *Physospermopsis*. And we mapped some valuable morphological characteristics of Physospermopsis on phylogenetic tree, including leaves, bracts and bracteoles, ribs of fruits. The BI and ML trees were constructed for analysis of interspecific relationships within *Physospermopsis* using ITS and plastid datasets from the 13 *Physospermopsis* populations we sampled, one *Physospermopsis* species and the two *Pleurospermum* species downloaded from NCBI. Detailed information on the investigated taxa can be found in the Appendix 1.

## Results

### Morphological characteristics of Physospermopsis

**Figure 1.**
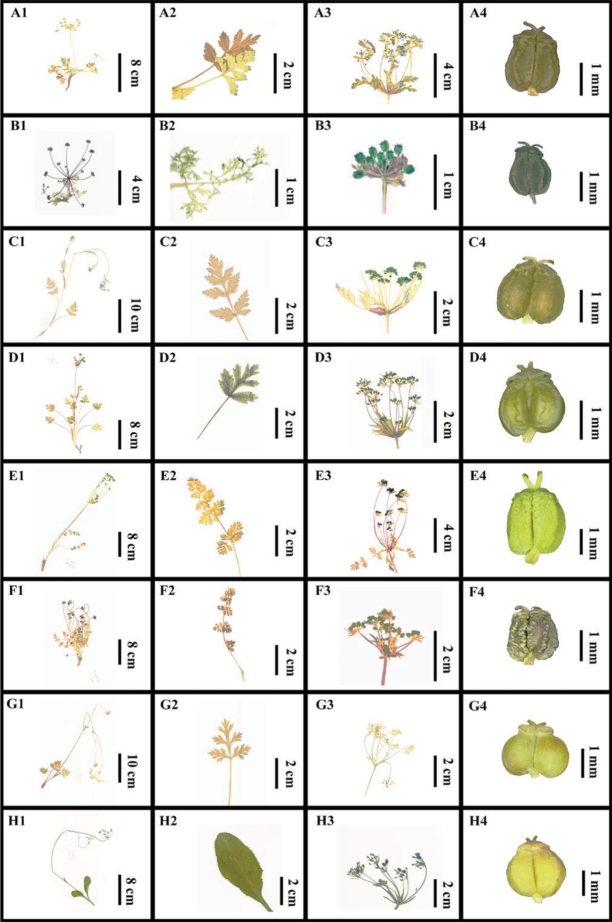
Morphological characters of *Physospermopsis***A1–H1** habit **A2–H2** basal leaf **A3–H3** umbel **A4–H4** mericarps **A1** habit of *P. shaniana***B1** habit of *P. nana***C1** habit of *P. muliensis***D1** habit of *P. rubrinervis***E1** habit of *P. obtusiuscula***F1** habit of *P. kingdon-wardii***G1** habit of *P. delavayi***H1** habit of *P. alepidioides***A2** basal leaf of *P. shaniana***B2** basal leaf of *P. nana***C2** basal leaf of *P. muliensis***D2** basal leaf of *P. rubrinervis***E2** basal leaf of *P. obtusiuscula***F2** basal leaf of *P. kingdon-wardii***G2** basal leaf of *P. delavayi***H2** basal leaf of *P. alepidioides***A3** umbel of *P. shaniana***B3** umbel of *P. nana***C3** umbel of *P. muliensis***D3** umbel of *P. rubrinervis***E3** umbel of *P. obtusiuscula***F3** umbel of *P. kingdon-wardii***G3** umbel of *P. delavayi***H3** umbel of *P. alepidioides***A4** mericarps of *P. shaniana***B4** mericarps of *P. nana***C4** mericarps of *P. muliensis***D4** mericarps of *P. rubrinervis***E4** mericarps of *P. obtusiuscula***F4** mericarps of *P. kingdon-wardii***G4** mericarps of *P. delavayi***H4** mericarps of *P. alepidioides*.

Through observations in the field, the most important characteristic to identify *Physospermopsis* species was prominent bracts and bracteoles. *Physospermopsis shaniana*, *P. nana*, *P. muliensis*, *P. rubrinervis*, *P. obtusiuscula* and *P. kingdon-wardii* usually have leaf-like bracts and bracteoles (Fig. 1A3–F3). While *P. alepidioides* and *P. delavayi* possess lanceolate or oblong bracts and bracteoles with a 2–3-lobed apex and dark purple margin (Fig. 1G3, H3). Furthermore, leaf shape varies with species and can be obovate-lanceolate (e.g. *P. alepidioides*), triangular (e.g. *P. rubrinervis*), obovate-orbicular (e.g. *P. delavayi*) or linear-lanceolate (e.g. *P. nana*) segments (Fig. 1B2, D2, G2, H2). Besides, the leaves of *P. kingdon-wardii* and *P. obtusiuscula* are 2-pinnate and ovate-oblong, and have 2–6 pairs of ovate pinnae with pinnatisect margin. *Physospermopsis muliensis* and *P. shaniana* possess 3–5 pairs pinnae with pinnatifid margin, narrowly winged petioles and narrow and purple-red sheaths. Fruit morphology was recorded prior to alcohol preservation because the alcohol altered the color slightly (as is seen in photographs). The fruits of *Physospermopsis* were emerald green or chartreuse, ovoid to broadly ovoid, and typically had a slightly cordate base, a gradually narrowed and laterally flattened apex, with filiform or prominent ribs. Fruit shape and size of all *Physospermopsis* species were similar except that *P. kingdon-wardii* had fruit half the size of other species and very prominent and sinuate ribs. *Physospermopsis nana* and *P. muliensis* fruits had relatively prominent and filiform ribs, but *P. muliensis* fruits had scattered warts especially on the ribs and *P. nana* had smaller fruit. *Physospermopsis obtusiuscula* fruits were ovoid with narrowly winged and sinuolate ribs. The fruit of *P. delavayi* had an obvious cordate base, and filiform and less prominent ribs. *Physospermopsis alepidioides*, *P. rubrinervis* and *P. shaniana* had ovoid, verucose fruits with prominent ribs, but *P. alepidioides* did not have a cordate base, while the other two species had a slightly cordate base. The fruit of *P. shaniana* had many small warts distinguishing it from *P. rubrinervis*. For easy reading and comparison, the main morphological characteristics were listed in Table 1.

**Table 1.** The morphological characteristics of eight *Physospermopsis* species.

Taxa	Bracts	Bracteoles	Fruits	Leaf shape	Ribs	Stems	Umbels
Physospermopsis delavayi	lanceolate or oblong	lanceolate	broadly ovoid, with obvious cordate base	winged, obovate-orbicular	filiform	branched above	7–13
*P. alepidioides*	lanceolate or oblong	ovate-lanceolate,entire	ovoid, with obscure cordate base	entire, obovate-lanceolate	prominent	branched above	5–14
*P. muliensis*	leaf-like	lanceolate, entire	broadly ovoid or ovoid	narrowly winged, pinnatifid	relatively prominent, with scattered warts	branched above, slender	7–18
*P. rubrinervis*	leaf-like	leaf-like, with purplish margin	ovoid, with slightly cordate base	triangular, with purple-red nerves	prominent	branched above, dark purple	6–17
*P. shaniana*	leaf-like	leaf-like, with purplish margin	ovoid, with slightly cordate base	narrowly winged, pinnatifid	prominent,with small warts	branched at the base, reduced	6–15
*P. obtusiuscula*	leaf-like	ovate-oblong	ovoid to broadly ovoid	ovate-oblong, pinnatisect	with narrowly winged, sinuolate	branched at the base, dark purple-green	5–20
*P. kingdon-wardii*	leaf-like	leaf-like, with purplish margin	broadly ovoid	ovate-oblong, pinnatisect	prominent, sinuate, with sparse minute warts	reduced, often acaulescent	5–11
*P. nana*	leaf-like	leaf-like, with membranous margin	broadly ovoid	linear-lanceolate	prominent, narrowly sinuolate-winged	reduced, slender	4–13

### Phylogenetic analyses

**Figure 2.**
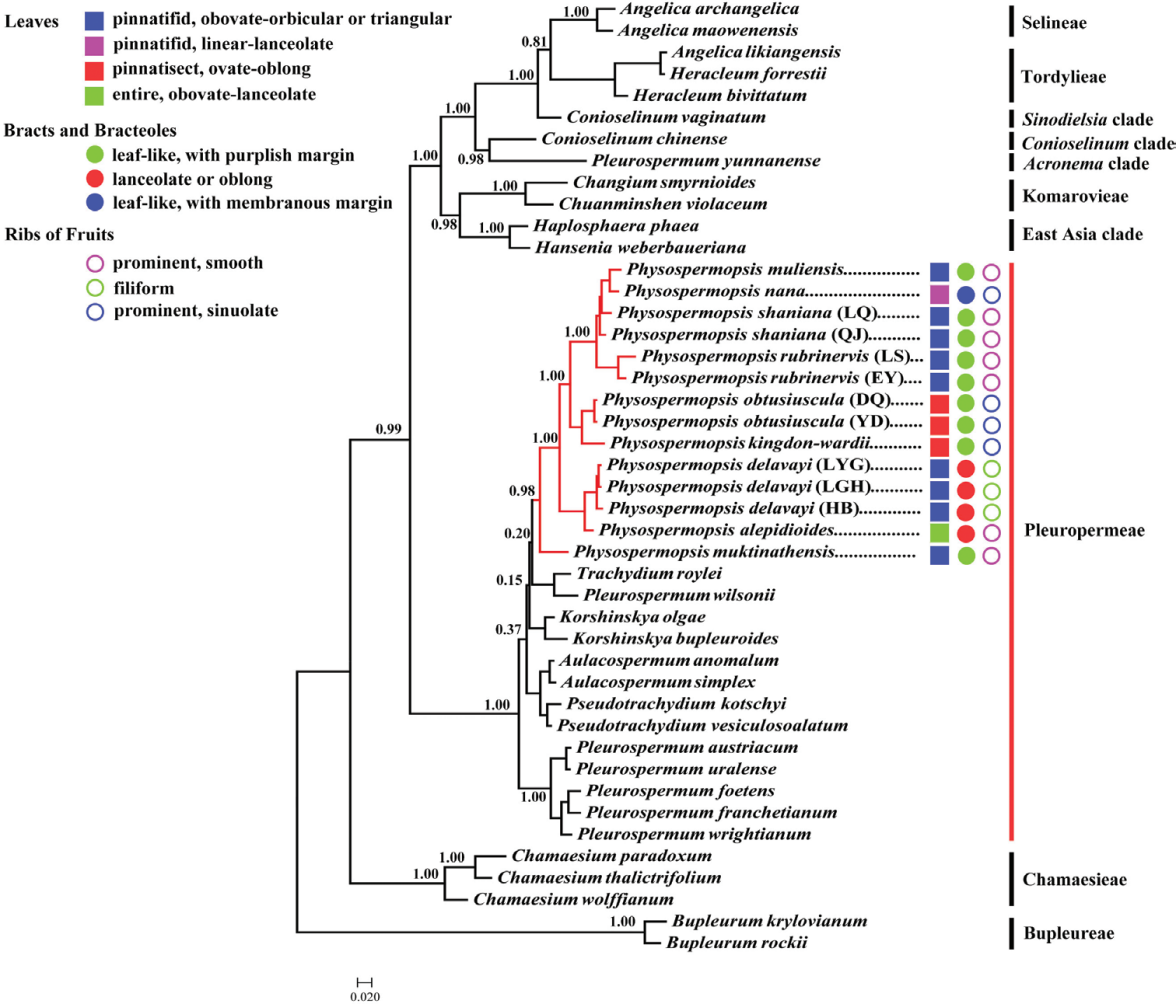
Bayesian tree inferred from the analysis of the 44 samples of ITS data. Branch lengths are proportional to the amount of character changes, scale = 0.02 substitutions per character. The tree is rooted with *Bupleurum*. The names of the clades identified are those of Zhou et al. (2008, 2009).

Through comprehensive sampling, the ITS analyses indicated that the 13 populations of *Physospermopsis* we sampled and *P. muktinathensis* M.A.Farille & S.B.Malla (1985: 512) formed an individual clade. *Physospermopsis* was confirmed to be a monophyletic group and nested in Pleurospermeae. *Trachydium roylei* Lindl. (1835: 232) and *Pl. wilsonii* H.Boissieu (1906: 433) were the closest relatives of *Physospermopsis* (Fig. 2).

**Figure 3.**
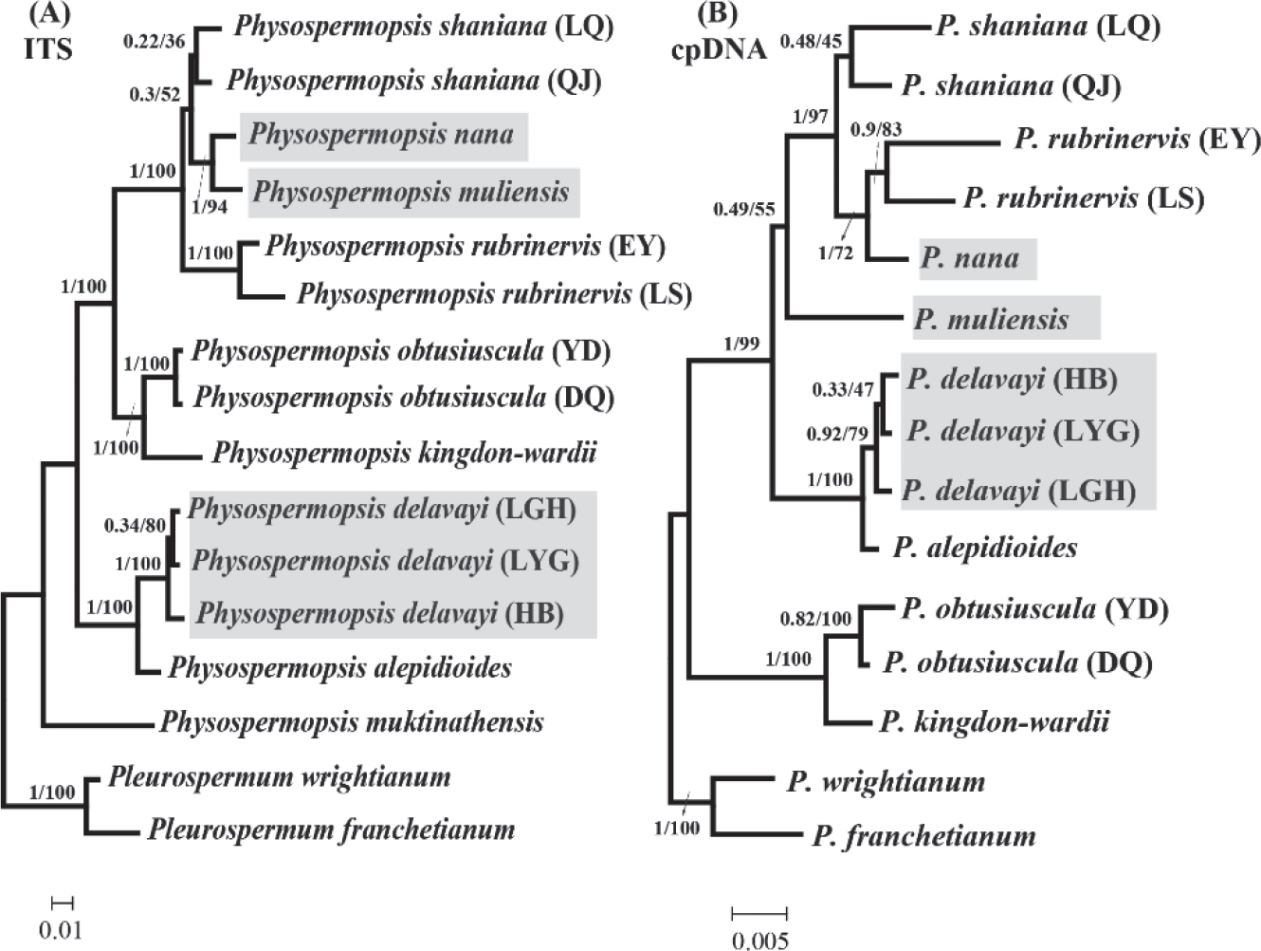
Bayesian trees of *Physospermopsis* and its related genus inferred from ITS (**A**) and plastid *rpl16*+*rps16* (**B**). Values on the branches indicate their support (Bayesian posterior probability/ Maximum-likelihood bootstrap). Branch lengths are proportional to the amount of character changes, scale = 0.01 (**A**), 0.005 (**B**) substitutions per character.

The ITS dataset tree topologies generated from BI and ML analyses were consistent. Therefore, only the BI tree with posterior probabilities (PP, 0–1) and bootstrap support values (BS, 0–100%) is illustrated in Fig. 3A. The first to differentiate from *Physospermopsis* was *P. muktinathensis*, which is distributed in Nepal. Three populations of *P. delavayi* and one of *P. alepidioides* united as a strongly supported (BI–PP = 1; ML–BS = 100%) group. *Physospermopsis obtusiuscula* was supported as a sister group to *P. kingdon-wardii* (BI–PP = 1; ML–BS = 100%). *Physospermopsis rubrinervis*, *P. muliensis*, *P. nana* and *P. shaniana* were allied in all trees (BI–PP = 1; ML–BS = 100%). However, clear interspecific relationships between *P. rubrinervis*, *P. muliensis*, *P. nana* and *P. shaniana* were not strongly supported by ML or BI analyses.

The cpDNA dataset tree topologies inferred by BI and ML analyses were consistent (Fig. 3B). However, results of a partition homogeneity test for the ITS and cpDNA datasets indicated that these genomes provide significantly different phylogenetic estimates. The taxa involved in this conflict are highlighted in Fig. 3. There was no chloroplast data for *P. muktinathensis*. The first to differentiate were *P. obtusiuscula* and *P. kingdon-wardii* (BI–PP = 1; ML–BS = 99%). The relationships of the three *P. delavayi* populations differed from the ITS dataset tree topology, although this cpDNA dataset relationship was not strongly supported. The cpDNA dataset tree topologies indicated that LYG population was closer to the HB population (BI–PP = 0.33; ML–BS = 47%), while LYG was closer to LGH in the ITS dataset tree topologies (BI–PP = 0.34; ML–BS = 70%). Additionally, the relationships between *P. rubrinervis*, *P. muliensis*, *P. nana* were not consistent with the ITS tree, where *P. nana* allied with *P. rubrinervis* in the cpDNA tree (BI–PP = 1; ML–BS = 72%), whereas *P. nana* allied with *P. muliensis* in the ITS tree (BI–PP = 1; ML–BS = 94%).

## Discussion

### The Phylogenetic position of *Physospermopsis* and relationship between *Physospermopsis* and *Pleurospermum*

*Physospermopsis* is monophyletic. The reasons for previous designations as a polyphyletic genus were likely attributable to the misidentification of several species (e.g. *P. rubrinervis*, *P. kingdon-wardii*, *P. cuneata*). Besides, *P. cuneata* is a poorly known species and unusual within the genus for its lack of conspicuous bracts and bracteoles, and therefore the phylogenetic placement of it is highly controversial. However, the most recent consensus is that *P. cuneata* should not be placed in *Physospermopsis* (Zhou et al. 2008; Zhou et al. 2009; Pimenov 2017). So previous molecular studies only involved five *physospermopsis* species which were widely accepted; we added another three *physospermopsis* species in this study, including *P. alepidioides*, *P. obtusiuscula*, and *P. nana*. Evidence obtained through more precise checking of generic type, infrageneric types and extensive herbarium specimens, literature and field investigations, analyzing morphological characters, and ITS and cpDNA evidence. This comprehensiveness allows us to be confident that *Physospermopsis* is monophyletic and nested in Pleurospermeae. In addition, we propose that the *Physospermopsis* clade should be replaced by the East Asia Clade.

The molecular results indicated that *Physospermopsis* is closest to *Pleurospermum*. Morphologically, *Pleurospermum* usually possess numerous bracts and bracteoles with white scarious margins, conspicuous or obsolete calyx teeth, white or purple-red petals with clawed base and narrow apex, prominent, acute ribs (Pan and Watson 2005). However, we found that *Physospermopsis* differed from *Pleurospermum* by less prominent and even inconspicuous fruit ribs, and the bracts and bracteoles did not have white scarious margins, resulting in an obvious, diagnostic boundary between *Pleurospermum* and *Physospermopsis*. The closeness of the two genera is also evidenced in pollen morphology. Wang and Pu (1992) found *P. alepidioides* and *P. muliensis* pollen to be rhomboidal and similar to several *Pleurospermum* species whereas other *Physospermopsis* species (*P. rubrinervis* and *P. delavayi*) have more advanced rectangular types. In addition, Pan and Watson (2005) identified several *Physospermopsis* species (e.g. *P. obtusiuscula*) with morphological similarities to *Pleurospermum* species, including having long fruit ribs and bulgy fruit walls, while other species had flattening of fruit and reduced wall thickness. Consequently, *Physospermopsis* is sister to *Pleurospermum*.

### Interspecific relationships within *Physospermopsis*

**Figure 4.**
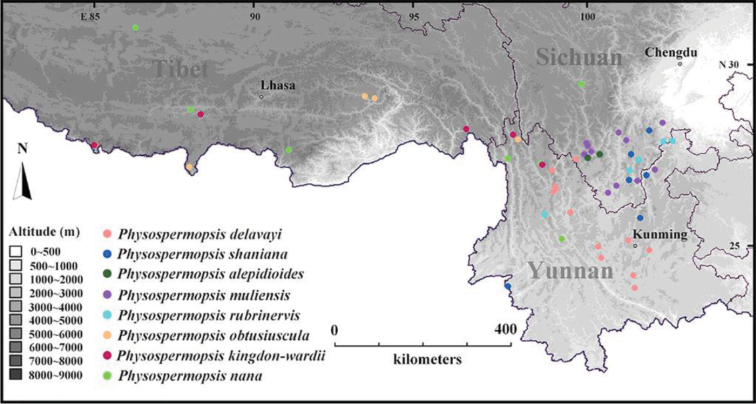
Geographic distribution of the eight Chinese *Physospermopsis* species in China. The altitude, scale, name of provinces and provincial capitals are also showed on the map.

The morphological characteristics mapped on the phylogenetic tree indicated that most closely related species have similar morphological characteristics. For example, *P. rubrinervis*, *P. muliensis* and *P. shaniana* are highly consistent on leaves, bracts and bracteoles, ribs on fruits (Fig. 2). Similarly, these species are the geographically sympatric species (Fig. 4). Resolution of the relationships between these species will only be achieved through continued studies, which may be difficult due to their geographic and morphological similarities. However, we can learn that *P. nana*, *P. rubrinervis*, *P. muliensis* and *P. shaniana* are the more advanced species in *Physospermopsis*. The morphological characters of *P. nana* are the most particular; these might be caused by hybridization with *Pleurospermum* species.

The interspecific relationships between certain species within *Physospermopsis* are evident based on the consistencies between ITS and cpDNA trees. For instance, *P. alepidioides* showed a close affinity to *P. delavayi* in phylogenetic tree, and they have similar bracts and bracteoles (entire or 2–3-lobed at apex, with dark purple margin) (Figs 1, 2). However, differing leaf shapes can be used to easily distinguish these two species because *P. alepidioides* has an undivided leaf with sparsely serrated margin (Fig. 1H2) and *P. delavayi* has a pinnate leaf (Fig. 1G2). *Physospermopsis kingdon-wardii* is sister to *P. obtusiuscula*, which is consistent with their geographic closeness. *Physospermopsis kingdon-wardii* appears more morphologically similar to *P. obtusiuscula* (including the leaves, bracts and bracteoles), but differs in its reduced stem, small stature and small fruits with prominent and sinuate ribs (Fig. 1).

The topologies of the ITS and cpDNA trees differed in the positioning of *P. delavayi*, *P. muliensis* and *P. nana* (Fig. 3). This inconsistency between nrDNA ITS and cpDNA data has been reported in some studies of Apiaceae (Lee and Downie 2006; Zhou et al. 2008, 2009; Spalik et al. 2009; Bone et al. 2011; Yi et al. 2015; Panahi et al. 2018). This difference generally has been caused by incomplete lineage sorting, hybridization, homoplastic substitutions and introgression. Since we did not sample by lineage and execute gene flow analysis, what caused the inconsistency cannot be determined. Previous studies have indicated that Pleurospermeae occupies a relative position in the base of the Apioideae (Zhou et al. 2008, 2009; Downie et al. 2010), the differentiation time should be earlier. Thus, for *Physospermopsis*, we infer the more effective reason for the inconsistency between nrDNA ITS and cpDNA data is hybridization. A further study based on widely sampling and deeper analysisis needed. However, several diagnostic characteristics can be utilized in the field and laboratory to separate them. *P. rubrinervis* can easily be recognized by purple-red nerves on the leaves, bracts and bracteoles with purple-red margin (Fig. 1). *Physospermopsis muliensis* possesses a slender, branched stem and narrowly winged basal petioles with narrow sheaths (Fig. 1). *Physospermopsis nana* has bracts and bracteoles with white scarious margins and linear-lanceolate segments with membranous-margined sheaths (Fig. 1). The stem of *P. shaniana* was reduced and branched at the base, and had prominent bracts 1–2-pinnate with developed, broad sheaths (Fig. 1).

## Taxonomy

### Chinese *Physospermopsis* species

#### Physospermopsis

Taxon classificationPlantae

H.Wolff (1925: 276)

C0DABFC0-2139-53CA-8E17-3DB3EA04DDC1

##### Type.

*Physospermopsis delavayi* H.Wolff (1925: 278)

### Key to the Chinese *Physospermopsis* species

**Table:** 

1	Leaves entire, margin sparsely serrate	***P. alepidioides***
–	Leaves pinnate or pinnatifid	2
2	Stems reduced, sometimes acaulescent	3
–	Stems developed	5
3	Bracteoles margin membranous	***P. nana***
–	Bracteoles margin purplish	4
4	Fruits ovoid, with slightly cordate base; ribs prominent, with small warts	***P. shaniana***
–	Fruits broadly ovoid; ribs prominent and sinuate, with sparse minute warts	***P. kingdon-wardii***
5	Nerves of leaves purple-red	***P. rubrinervis***
–	Nerves of leaves green	6
6	Bracts lanceolate or oblong; ribs filiform	***P. delavayi***
–	Bracts leaf-like; ribs prominent	7
7	Bracteoles ovate-lanceolate,entire; ribs with scattered warts	***P. muliensis***
–	Bracteoles ovate-oblong; ribs with narrowly winged, sinuolate	***P. obtusiuscula***

#### 1. Physospermopsis alepidioides

Taxon classificationPlantae

(H.Wolff et Hand.-Mazz.) R.H.Shan, 1941: 187

2797F21D-1AD8-5CE5-8BE0-9F4EA8FAA97F

≡ Haploseseli alepidioides H.Wolff et Hand.-Mazz., 1933: 722

##### Type.

China. Sichuan: Yanyuan County, 2700–2800 m, 7 Oct 1914, *Handel-Mazzetti 5562* (holotype: WU [WU0060774]).

##### Diagnostic characters.

*Physospermopsis alepidioides* usually possesses an entire leaf blade with a sparsely serrated margin. The shape of the entire leaf segment is an obvious diagnostic characteristic to distinguish it from other *Physospermopsis* species. The stem of it is velutinous.

##### Distribution.

Endemic to China, Sichuan (Fig. 4).

##### Habitat.

*Physospermopsis alepidioides* usually occurs in open forests and grasslands.

##### Additional specimens examined.

**China. Sichuan Province**: Muli County, Hetaowan, 2300 m, 8 Aug 2019, *X.R.Xu XXR2019080801* (SZ); Muli County, Hetaowan, 2250 m, 23 Dec 1982, *Y.B.Yang & Y.L.Cao 400* (CDBI); Muli County, Liziping, 21 Sep 2011, *X.G.Ma m11092101* (SZ); unknown locality, 2650 m, 19 Jul 1983, *Anonymous 22* (HITBC); Yanyuan County, Mt. Huolu, 3950 m, 22 Jul 1983, *Anonymous 25* (HITBC).

#### 2. Physospermopsis rubrinervis

Taxon classificationPlantae

(A.R.Franchet) C.Norman, 1938: 231

64822BFE-1082-5DA6-9C82-766A751968DB

≡ Trachydium rubrinerve A.R.Franchet, 1894: 112
≡ Pleurospermum rubrinerve (A.R.Franchet) M.Hiroe, 1979: 747

##### Type.

China. Yunnan: Eryuan County, Mt. Luoping, 3200 m, 31 Aug 1888, *Delavay 3235* (holotype: P [P00245453]; lectotype, designated by Pimenov 2017, pg. 188: P [P00245453] ; isolectotypes: K [K001235378], P [P00245454, P00834665]).

##### Diagnostic characters.

*Physospermopsis rubrinervis* usually possesses dark purple, sparsely branching stems. The basal blade is ovate to broadly ovate in outline, having almost purple-red nerves.

##### Distribution in China.

Sichuan, Yunnan (Fig. 4).

##### Distribution outside China.

India, Nepal.

##### Habitat.

This species grows in the forest edge or rhododendron shrubs at an elevation of 2800–4800 m.

##### Additional specimens examined.

**China. Sichuan Province**: Yanbian County, Yankou xiang, 3150 m, 20 Sep 2002, *X.F.Gao*, *Y.L.Peng & G.Sun 3753* (PE); Meigu County, Ligou xiang, 3600 m, 5 Aug 1959, *1591* (SM); Butuo County, Wuke pasture, 3500 m, 1 Jul 1976, *Vegetation expedition 13827* (CDBI); Dukou County, Mt. Dahei, 1400 m, 18 Jun 1983, *Qinghai-Tibet Expedition 11231* (KUN); Puge County, 9 Aug 1960, *Anonymous 25099* (SM); **Yunnan Province**: Eryuan County, Mt. Luoping, 3200 m, 17 Aug 2019, *X.R.Xu XXR2019081701* (SZ); Lushui County, 3000 m, 18 Oct 2019, *X.L.Guo G19101802* (SZ); unknown locality, 21 Sep 1959, *S.G.Wu 2715* (KUN).

#### 3. Physospermopsis kingdon-wardii

Taxon classificationPlantae

(H.Wolff) C.Norman, 1938: 231

5658104D-38FB-58B3-AE5B-8258CFE46DF9

≡ Trachydium kingdon-wardii H.Wolff, 1929: 124
≡ Pleurospermum kingdon-wardii (H.Wolff) M.Hiroe,1979: 747

##### Type.

China. Yunnan: A-tun-tsi, screes, turf, 14000 ft. (ca. 4267 m), 7 Aug 1913, *Kingdon-Ward 992* (lectotype: E [E00000221]).

##### Diagnostic characters.

*P. kingdon-wardii* is similar to *P. obtusiuscula* in shape of basal leaves, but the stem of *P. kingdon-wardii* is reduced. The fruits are smaller than other species, and the immature fruits sometimes have sparse minute warts. Additionally, the ribs are prominent, often sinuate.

##### Distribution in China.

Tibet, Yunnan (Fig. 4).

##### Distribution outside China.

Bhutan, Nepal, Sikkim.

##### Habitat.

*Physospermopsis kingdon-wardii* usually grows in alpine meadows or scrubs at about 3900 m elevation.

##### Additional specimens examined.

**China. Tibet Province**: Nyalam County, 4000 m, 24 Aug 2019, *X.L.Guo G19082407* (SZ); Bainang County, 4580 m, 24 Aug 1988, *Anonymous 8* (CDBI); Zayü County, 4180 m, 27 Sep 1982, *Qinghai-Tibet Expedition 10772* (PE); Zayü County, 4370 m, 31 Aug 2003, *X.F.Gao*, *W.G.Tu*, *H.He & Y.K.Qiao 6745* (CDBI); **Yunnan Province**: Dêqên County, 4300 m, 28 Sep 1981, *L.R.Xu 129* (WUK); Dêqên County, 3900 m, 18 Aug 1940, *K.M.Feng 6746* (PE); Dêqên County, 4500 m, 23 Sep 1986, *H.Sun & Z.G.Qian 751* (KUN); Zhongdian County, 4300 m, 2 Oct 1986, *H.Sun & Z.G.Qian 0980* (KUN).

#### 4. Physospermopsis obtusiuscula

Taxon classificationPlantae

(DC.) C.Norman, 1938: 231

1F62F899-E5DE-5F7C-936E-C0778EE460C9

≡ Hymenolaena obtusiuscula DC., 1830: 246
≡ Trachydium obtusiusculum (DC.) C.B.Clarke, 1879: 673
≡ Pleurospermum obtusiusculum (DC.) M.Hiroe, 1979: 741
≡ Aulacospermum obtusiusculum (DC.) A.R.Naqshi, U.Dhar et P.N.Kachroo, 1995: 107

##### Type.

Nepal. “Ad Gossain-Than Nepalensium, Wallich [543]” (lectotype: G-DC; isolectotypes: BM [BM000622303, BM000944782], K [K000697363], K-WALLICH, LE).

##### Diagnostic characters.

*Physospermopsis obtusiuscula* sometimes is flushed. The stems are dark purple-green, simple, and occasionally branched at the base. The fruit ribs are narrowly winged and sinuolate, which is a unique character in *Physospermopsis*.

##### Distribution in China.

Sichuan, Tibet, Yunnan (Fig. 4).

##### Distribution outside China.

Bhutan, India, Nepal, Sikkim.

##### Habitat.

*Physospermopsis obtusiuscula* grows in shrubs or grassland at an elevation of ca. 4000 m.

##### Additional specimens examined.

**China. Sichuan Province**: Xiangcheng County, 3900 m, 9 Aug 1981, *Qinghai-Tibet Expedition 3986* (PE); Xiangcheng County, 9 Aug 1981, *Qinghai-Tibet Expedition 3942* (PE); **Tibet Province**: Yadong County, 3500 m, 20 Aug 2019, *X.L.Guo G19082009* (SZ); Nyingchi County, 3400 m, 8 Aug 1983, *B.S.Li & S.Z.Cheng 6199* (PE); Nyingchi County, Mt. Shergyla, 3346 m, 13 Oct 2009, *J.Luo*, *S.L.Wang & G.Y.Wang LiuJQ-09XZ-388* (KUN); Nyingchi County, Mt. Shergyla, 3346 m, 13 Oct 2009, *J.Luo*, *S.L.Wang & G.Y.Wang LuoJian-ZX-0938* (PE); Yadong County, 3980 m, 14 Sep 1974, *Qinghai-Tibet Expedition 74-2505* (PE); Yadong County, 4000 m, 12 Sep 1974, *Qinghai-Tibet Expedition 2416* (PE); Nyalam County, 3800 m, 2 Sep 1972, *1736* (PE); Zayü County, 4300 m, 26 Sep 1982, *Qinghai-Tibet Expedition 10635* (PE); **Yunnan Province**: Dêqên County, Baimaxueshan, 4100 m, 15 Aug 2019, *X.R.Xu XXR2019081502* (SZ); Gongshan County, Dulongjiang, 2900 m, 15 Sep 1938, *T.T.Yü 20274* (PE); Gongshan County, Dulongjiang, 3800 m, 9 Aug 1938, *T.T.Yü 19829* (PE).

#### 5. Physospermopsis muliensis

Taxon classificationPlantae

R.H.Shan et S.L.Liou, 1979: 105

43C4D771-1AAE-5D6E-8A60-DF7E2C6DD345

##### Type.

China. Sichuan: Muli County, 4000 m, 20 Oct 1937, *T.T.Yu 14579* (holotype: PE [P01432306]).

##### Diagnostic characters.

*Physospermopsis muliensis* usually possesses branching stems, ovate-oblong leaf blades, narrow sheaths, leaf-like bracts, lanceolate bracteoles, and ovoid fruits with filiform ribs with sparse scattered warts. Basal and lower petioles are narrowly winged.

##### Distribution.

Endemic to China, Sichuan (Fig. 4).

##### Habitat.

*Physospermopsis muliensis* usually grows in wet grasslands at 2500–4100 m elevation.

##### Additional specimens examined.

**China. Sichuan Province**: Muli County, Kangwuliangzi, 3800 m, 9 Aug 2019, *X.R.Xu XXR2019080903* (SZ); Muli County, 3900 m, 20 Oct 1981, *L.R.Xu 0187* (WUK); unknown locality, 3600 m, 29 November 2005, *Anonymous 416* (PE); unknown locality, 2580 m, 28 November 2005, *Anonymous 418* (PE); unknown locality, 3650 m, 29 November 2005, *Anonymous 419* (PE); Muli County, 3800 m, 21 Sep 1955, *Anonymous 267* (PE); Muli County, 3620 m, 15 Oct 1982, *Y.B.Yang & G.Yao 174* (CDBI); Muli County, 3200 m, 3 Sep 1978, *Q.S.Zhao*, *K.H.Mou & Y.B.Yang 8395* (CDBI); Muli County, 3650 m, 25 Oct 1986, *Y.J.Li et al. 987* (CDBI); Muli County, 3150 m, 25 Oct 1982, *Vegetation expedition 29926* (CDBI); Muli County, 3900 m, 22 Aug 1983, *39* (HITBC); Dukou County, 21 Sep 1978, *412* (SM); Jinyang County, Mt. Shizi, 4000 m, 18 Aug 1978, *683* (SM); Mianning County, Juexingou, 3000 m, 11 Oct 1978, *Mianning expedition 667* (SM); Ningnan County, 3700 m, 24 Aug 1978, *Ningnan expedition 547* (SM); Dukou County, Yanbian, 28 Sep 1978, *Anonymous 435* (SM); Meigu County, 3700 m, 18 Aug 1979, *Anonymous 594* (SM); Yanbian County, 28 Sep 1978, *Anonymous 475* (SM); Zhaojue County, 4040 m, 13 Jul 1979, *Plants census expedition 179* (SM); Xide County, 29 Jun 1979, *Anonymous 495* (SM); Mabian County, 3400 m, 1 Jul 1978, *Mabian expedition 798* (SM); Muli County, 3900 m, 2 Aug 1978, *Muli expedition 577* (SM); Muli County, Changhaizi, 3669 m, 7 Oct 2009, *Z.L.Nie*, *Y.Meng & T.Deng SunH-07ZX-2330* (KUN).

#### 6. Physospermopsis shaniana

Taxon classificationPlantae

Z.Y.Wu et F.D.Pu, 1993: 1285

E2DC4568-2DD6-5EFE-8662-D1DE8FE4F48D

≡ Trachydium forrestii Diels, 1912: 291
≡ Physospermopsis forrestii (Diels) C.Norman, 1938: 231
≡ Pleurospermum forrestii (Diels) M.Hiroe, 1958: 123

##### Type.

China. Yunnan: Lijiang range, shady, grassy openings in pine forests on the eastern flank, Aug 1906, *Forrest 2855* (lectotype, designated by Pimenov 2017, pg. 188: E [E00000219]; isolectotype: P [P00245432]).

##### Diagnostic characters.

*Physospermopsis shaniana* usually possesses 2-pinnate/pinnatifid, ovate-oblong leaf blades, broad sheaths, broadly ovoid fruits, white petals, and leaf-like, 2-pinnate bracts. The pinnae are subsessile with pinnatifid margin. The stems of *P. shaniana* are reduced and branched at the base. The branches are longer than the main stem.

##### Distribution in China.

Sichuan, Tibet, Yunnan (Fig. 4).

##### Distribution outside China.

Myanmar.

##### Habitat.

*Physospermopsis shaniana* usually grows in pasture and grassy slopes.

##### Additional specimens examined.

**China. Sichuan Province**: Meigu County, Kongmingzhai, 3600 m, 5 Aug 1959, *Anonymous 1591* (KUN); Zhaojue County, Jiefanggou, 3200 m, 9 Jul 1976, *Vegetation expedition 12928* (PE); **Yunnan Province**: Qiaojia County, Yaoshan, 3200 m, 14 Jul 2019, *X.R.Xu XXR2019071404* (SZ); Luquan County, Jiaozixueshan, 3700 m, 17 Jul 2019, *X.R.Xu XXR2019071701* (SZ); Dongchuan County, Huizedahai, 3400 m, 30 Jul 1964, *Northeast Yunnan Expedition 434* (KUN); Qiaojia County, *G.M.Yang SCSB-W-1237* (KUN); Zhenkang County, 8 Jul 1938, *T.T.Yu 17120* (PE); Zhenkang County, snow range, 3450 m, 4 Aug 1938, *T.T.Yu 17174* (PE); Dongchuan County, Mubanghai, 3360 m, 16 Aug 1964, *Northeast Yunnan Expedition 822* (LBG,KUN); Zhaotong County, 24Gang, 2300 m, 10 Aug 1974, *Anonymous 234* (KUN); Qiaojia County, Yaoshan, 3100 m, 16 Jul 1973, *B.X.Sun 1017* (KUN).

#### 7. Physospermopsis delavayi

Taxon classificationPlantae

(A.R.Franchet) H.Wolff, 1925: 278

8A7A5242-A006-5E20-A9AC-A763FB810762

≡ Arracacia delavayi A.R.Franchet, 1894: 115
≡ Pleurospermum delavayi (A.R.Franchet) M.Hiroe, 1958: 120

##### Type.

China. Yunnan: Mosuoying, Yangyushan, 15 Sep 1885, *Delavay 2017* (lectotype, designated by Pimenov and Kljuykov 2000c, pg. 537: P [P00245424]).

##### Diagnostic characters.

*P. delavayi* usually possesses a conspicuously winged rachis and yellow-green, round fruits. The bracts are usually smaller than other species of *Physospermopsis*. The basal leaves are obovate to obovate-orbicular with incised-serrate or lobed margin or cuneate with partite margin.

##### Distribution.

Endemic to China, Hunan, Sichuan, Yunnan. (Fig. 4).

##### Habitat.

*Physospermopsis delavayi* prefers to grow in the pine forest or open grasslands.

##### Additional specimens examined.

**China. Sichuan Province**: Yanyuan County, Lugu Lake, 3200 m, 11 Aug 2019, *X.R.Xu XXR2019081102* (SZ); **Yunnan Province**: Lijiang County, Mt. Yulong, 3000 m, 13 Aug 2019, *X.R.Xu XXR2019081305* (SZ); Shangri-La, 3200 m, 18 Jul 2018, *X.L.Guo G18071802* (SZ); Lijiang County, Ganhaizi, 3100 m, 14 Feb 1968, *Anonymous s.n.* (HITBC); Lijiang County, Baishuihe, 2980 m, 4 Aug 1962, *Anonymous s.n.* (HITBC); Shuangbai County, Tuodian, 6 Oct 1958, *S.Q.Huang 0217* (LBG); Chuxiong County, Baomanjie, 19 Sep 1958, *S.Q.Huang 035* (LBG); Jianshui County, Yangjieba, 17 Mar 1941, *S.E.Liu 018312* (PE); Eryuan County, Chaijiaying, Mt. Longtou, 21 Jul 1929, *R.C.Qin 23338* (PE); unknown locality, *Anonymous 3365* (PE); unknown locality, *M.Chen 2460* (KUN); *W.R.He 2381* (KUN); Fumin County, Mt. Laoqing, 2300 m, 19 Oct1964, *B.Y.Qiu 596086* (KUN); Yiliang County, Qixingcun, 27 Aug 1975, *B.Y.Qiu 60727* (KUN); Xicheng County, Qingshuihe, 1900 m, 12 Oct1982, *B.T.Yue 2088* (KUN); Shangri-La County, Habacun, 2800 m, 3 Aug 1962, *A.L.Zhang 100750* (KUN); Lijiang County, Mt. Yulong, Baishuihe, 3000 m, 11 Jul 1962, *A.L.Zhang*, *S.W.Yu 100919* (KUN); Lijiang County, Yuhucun, 29 Aug 2010, *Z.X.Wang WZX2010082911* (SZ); Lijiang County, Yuhucun, 31 Aug 2010, *Z.X.Zhang WZX2010083102* (SZ); unknown locality, 29 Aug 2010, *P.Gao*, *S.Liu 10829-6* (SZ); unknown locality, 17 Sep 1938, *12366* (PEY); 22 Sep 1919, *K.K.TSOONG 387* (PEY).

#### 8. Physospermopsis nana

Taxon classificationPlantae

(A.R.Franchet) M.G.Pimenov et E.V.Kljuykov, 2000c: 538

8687EE7C-4892-51AC-812B-05DFBE8754AE

= Pleurospermum nanum A.R.Franchet, 1894: 140. Type: CHINA. Yunnan: Dali County, Mt. Cang, 25 Sep 1884, *Delavay 197* (syntypes: P [P00834544, P00834545]).

##### Type.

China. Yunnan: Mt. Cang, 4000 m, 30 Aug 1889, *Delavay 4066* (lectotype, designated by Pimenov and Kljuykov 2000c, pg. 538: P [P00834546]; isolectotype, designated by Pimenov and Kljuykov 2000c, pg. 538: P [P00834547]).

##### Diagnostic characters.

*Physospermopsis nana* usually possesses reduced stem, membranous-margined sheaths, and leaf-like bracts. The bracteoles are pale green with whitish margin in lower half. The ultimate segments are linear-lanceolate. The characters mentioned above are sufficient to distinguish it from other *Physospermopsis* species.

##### Distribution.

Endemic to China, Sichuan, Tibet, Yunnan. (Fig. 4).

##### Habitat.

*Physospermopsis nana* usually grows on marshy meadows, under shrubs.

##### Additional specimens examined.

**China. Sichuan Province**: Yajiang County, 3800 m, 9 Aug 1979, *Yajiangdui 293* (SM); Muli County, Sanqu, 3600 m, 13 Sep 1983, *Qinghai-Tibet Expedition 14043* (KUN); **Tibet Province**: Cona County, 4561 m, 10 Aug 2015, *L.Wei & J.C.Hao 15544* (BNU); Lahsa County, 5200 m, 3 Sep 1965, *G.C.Xia & T.K.Mi 2610* (KUN); Gê’gyai County, 5200 m, 21 Aug 1976, *Qinghai-Tibet Expedition 8710* (KUN); Lahsa County, 6000 m, 1 Sep 1965, *Y.T.Zhang & K.Y.Lang 2412* (KUN); Gê’gyai County, 5185 m, 9 Sep 2017, *Y.He BNU2017XZ325* (BNU); Shigatse, 4645 m, 23 Aug 2017, *Y.He & D.H.Liu BNU2017XZ064* (BNU); Gar County, 4360 m, 31 Aug 2008, *J.H.Chen et al YangYP-Q-0122* (KUN). **Yunnan Province**: Lanping County, 4200 m, 18 Oct 2019, *X.L.Guo G19101802* (SZ); Lijiang County, Mt. Yulong, 4400 m, 18 Aug 1976, *Y.Q.He 049* (WUK); Dêqên County, Baima snow range, 4400 m, 14 Jul 1981, *Qinghai-Tibet Expedition 2774* (HITBC); Gongshan County, Mt. Nanwan, 3400 m, 22 Sep 1997, *9604* (KUN); Zhongdian County, 4190 m, 2 Oct 2005, *Z.D.Fang et al PL-130* (SABG); Lijiang County, Yuhu, 4000 m, 8 Sep 1955, *G.M.Feng 21475* (KUN); Lijiang County, 4100 m, 31 Aug 1963, *C.Z.Bao 20229* (KUN); Zhongdian County, 4200 m, 2 Aug 1986, *H.Sun & Z.G.Qian 991* (KUN); Lijiang County, snow range, 15 Sep 1940, *R.C.Qin 31043* (KUN); Zhongdian County, Mt. Haba, 4000 m, 31 Aug 1962, *Zhongdian Expedition 1687* (KUN); Zhongdian County, Haba snow range, 26 Aug 1939, *G.M.Feng 2215* (KUN); Lijiang County, Yulong, 4000 m, 31 Aug 1963, *C.Z.Bao 20226* (KUN); Lijiang County, 2600 m, 27 Jul 1937, *D.J.Yu 15374* (KUN); Lijiang County, Mt. Yulong, 3200 m, 26 Aug 1961, *R.L.Xiong & Y.F.Qi 612715* (KUN); Dali County, 20 Aug 1945, *H.C.Wang 4507* (KUN); unknown locality, 1963, *J.S.Yang 2374* (KUN); Zhongdian County, 3400 m, 28 Jun 2009, *Z.D.Fang G-297* (SABG); Dali County, Mt. Cang, 3460 m, 15 Oct 1990, *CLD-90* (PE).

## Conclusion

*Physospermopsis* is monophyletic and nested in Pleurospermeae, sister to *Pleurospermum*. Although the interspecific relationships within *Physospermopsis* were not well resolved and the positioning of species was unclear, the relationships of *P. alepidioides* and *P. delavayi*, *P. kingdon-wardii* and *P. obtusiuscula* are close. Diagnostic characteristics for distinguishing the species in the field and laboratory are provided for necessary morphological and molecular research in future *Physospermopsis* phylogenetic studies.

## Supplementary Material

XML Treatment for Physospermopsis

XML Treatment for Physospermopsis alepidioides

XML Treatment for Physospermopsis rubrinervis

XML Treatment for Physospermopsis kingdon-wardii

XML Treatment for Physospermopsis obtusiuscula

XML Treatment for Physospermopsis muliensis

XML Treatment for Physospermopsis shaniana

XML Treatment for Physospermopsis delavayi

XML Treatment for Physospermopsis nana

## Appendix 1

**Table:** Voucher details and GenBank accession numbers of taxa used in this study. A n-dash (–) indicates unavailable information; new sequences are in bold.

Taxa	Voucher	Locality	Genbank accession numbers		
			ITS	*rpl16*	*rps16*
*Angelica archangelica*	Downie 79 (ILL)	cult. University of Joensuu Botanical Garden, Finland	AH003539	AF094362	AF110536
*Angelica likiangensis*	200421 (NHW)	Lijiang, Yunnan, China	DQ263587	FJ385074	FJ385172
*Angelica maowenensis*	ZJ0582 (KUN)	Mt. Gongga, Sichuan, China	EU236157	FJ385075	FJ385173
*Aulacospermum anomalum*	19932275 (E)	cult. Royal Botanic Garden, Edinburgh, United Kingdom	AF008641, AF009120	AF094440	AF110558
*Aulacospermum simplex*	Dingelstedt & Sovetkina 367 23-VII-1927 (LE)	Kazakhstan	GQ379339	–	AF110557
*Bupleurum krylovianum*	ZJ0726 (KUN)	KaNaSi Lake, Xinjiang, China	FJ385035	FJ385082	FJ385180
*Bupleurum rockii*	J059 (KUN)	Ninglang, Yunnan, China	FJ385036	FJ385083	FJ385181
*Chamaesium paradoxum*	ZJ0560 (KUN)	Daocheng-Litang, Sichuan, China	EU236161	FJ385085	FJ385184
*Chamaesium thalictrifolium*	ZJ0607 (KUN)	Zhangla-Caowan, Sichuan, China	EU236162	FJ385086	FJ385185
*Chamaesium wolffianum*	ZJ0525 (KUN)	Shudu Lake, Yunnan, China	EU236163	FJ385087	FJ385186
*Changium smyrnioides*	J101 (KUN)	Jiangsu Institute of Botany, China	DQ517340	FJ385088	FJ385187
*Chuanminshen violaceum*	J105 (KUN)	Cangxi, Sichuan, China	FJ385040	FJ385089	FJ385188
*Conioselinum chinense*	Raiche 30046 (UC)	California, America	U78374	AF094421	GU395135
*Conioselinum vaginatum*	ZJ0731 (KUN)	KaNaSi Lake, Xinjiang, China	FJ385041	FJ385091	FJ385190
*Haplosphaera phaea*	ZJ0521 (KUN)	Shudu Lake, Yunnan, China	EU236167	FJ385096	FJ385194
*Hansenia weberbaueriana*	ZJ0697 (KUN)	KIB nursery, Yunnan, China	EU236180	FJ385115	FJ385212
*Heracleum bivittatum*	ZJ0611 (KUN)	MaoCountyg, Sichuan, China	EU236168	FJ385098	FJ385196
*Heracleum forrestii*	ZJ091032 (KUN)	Shangri-La, Yunnan, China	EU236169	FJ385099	FJ385197
*Korshinskya bupleuroides*	Pimenov et al. 106 (MW)	–	FJ489360, FJ489391	–	–
*Korshinskya olgae*	Pimenov et al. 228 (MW)	–	FJ489359, FJ489390	–	–
*Physospermopsis alepidioides*	XXR2019080801 (SZ)	Muli, Sichuan, China	**MT533355**	**MT542144**	**MT561011**
*Physospermopsis delavayi* (HB)	G18071802 (SZ)	Shangri-La, Yunnan, China	**MN658653**	**MN786490**	**MN786487**
*Physospermopsis delavayi* (LGH)	XXR2019081102 (SZ)	Lugu Lake, Sichuan, China	**MN658656**	**MN786488**	**MN786486**
*Physospermopsis delavayi* (LYG)	XXR2019081305 (SZ)	Lijiang, Yunnan, China	**MN658657**	**MN786489**	**MN786485**
*Physospermopsis kingdon-wardii*	G19082407 (SZ)	Nyalam, Tibet, China	**MN659655**	**MN786491**	**MN786484**
*Physospermopsis muktinathensis*	Pimenov & Kljuykov 22 (MW)	Annapurna, Nepal	FJ469961, FJ483500	–	–
*Physospermopsis muliensis*	XXR2019080903 (SZ)	Muli, Sichuan, China	**MT533356**	**MT542145**	**MT561012**
*Physospermopsis nana*	G19101802 (SZ)	Lanping, Yunnan, China	**MT542694**	**MT561018**	**MT561017**
*Physospermopsis obtusiuscula* (DQ)	XXR2019081502 (SZ)	Dêqên, Yunnan, China	**MT533361**	**MT542149**	**MT561016**
*Physospermopsis obtusiuscula* (YD)	G19082009 (SZ)	Yadong, Tibet, China	**MT533360**	**MT542148**	**MT561015**
*Physospermopsis rubrinervis* (EY)	XXR2019081701 (SZ)	Eryuan,Yunnan, China	**MN658654**	**MN786492**	**MN786483**
*Physospermopsis rubrinervis* (LS)	G19101802 (SZ)	Lushui, Yunnan, China	**MT533359**	**MT542143**	**MT561010**
*Physospermopsis shaniana* (LQ)	XXR2019071701 (SZ)	Luquan, Yunnan, China	**MT533357**	**MT542146**	**MT561014**
*Physospermopsis shaniana* (QJ)	XXR2019071404 (SZ)	Qiaojia, Yunnan, China	**MT533358**	**MT542147**	**MT561013**
*Pleurospermum austriacum*	Ghisa and Topa 2959 (MW)	–	FJ469962, FJ483502	–	–
*Pleurospermum foetens*	Chungtien 1181 (E)	Yunnan, China	FJ483482, FJ469943	AF094438	AF110559
*Pleurospermum franchetianum*	ZJ0573 (KUN)	Kangding, Sichuan, China	EU236198	FJ385137	FJ385232
*Pleurospermum uralense*	LQX031 (NAS)	Liaoning, China	JF977839	AF094439	AF110560
*Pleurospermum wilsonii*	ZJ0624 (KUN)	Hongyuan, Sichuan, China	EU236200	FJ385139	FJ385234
